# Sustainable Green Pavement Using Bio-Based Polyurethane Binder in Tunnel

**DOI:** 10.3390/ma12121990

**Published:** 2019-06-21

**Authors:** Chao Leng, Guoyang Lu, Junling Gao, Pengfei Liu, Xiaoguang Xie, Dawei Wang

**Affiliations:** 1 School of Physical Education, Harbin University, Harbin 150090, China; jwwkc207@hrbu.edu.cn; 2 School of Transportation Science and Engineering, Harbin Institute of Technology, Harbin 150090, China; lu@isac.rwth-aachen.de (G.L.); 18S132071@stu.hit.edu.cn (J.G.); xxg75@126.com (X.X.); 3 Institute of Highway Engineering, RWTH Aachen University, 52074 Aachen, Germany

**Keywords:** tunnel pavement, sustainable pavement material, polyurethane thin overlay, combustion properties, noise reduction, solar reflection

## Abstract

As a closed space, the functional requirements of the tunnel pavement are very different from ordinary pavements. In recent years, with the increase of requirements for tunnel pavement safety, comfort and environmental friendliness, asphalt pavement has become more and more widely used in long tunnels, due to its low noise, low dust, easy maintenance, and good comfort. However, conventional tunnel asphalt pavements cause significant safety and environmental concerns. The innovative polyurethane thin overlay (PTO) has been developed for the maintenance of existing roads and constructing new roads. Based on the previous study, the concept of PTO may be a feasible and effective way to enrich the innovative functions of tunnel pavement. In this paper, the research aims to evaluate the functional properties of PTO, such as noise reduction, solar reflection and especially combustion properties. Conventional asphalt (Open-graded Friction Course (OGFC) and Stone Mastic Asphalt (SMA)) and concrete pavement materials were used as control materials. Compared with conventional tunnel pavement materials, significant improvements were observed in functional properties and environmental performance. Therefore, this innovative wearing layer can potentially provide pavements with new eco-friendly functions. This study provides a comprehensive analysis of these environmentally friendly materials, paving the way for the possible application in tunnels, as well as some other fields, such as race tracks in stadiums.

## 1. Introduction

As a closed space, the functional requirements of the tunnel pavement are very different from ordinary pavements, e.g., a higher requirement on the reduction of the pavement noise, proper light reflection to ensure safety and save tunnel lighting energy, closure of the construction process, better pollutant discharge and air purification.

For a long time, cement concrete pavement has been widely used in tunnels, due to its long service life and better lighting effect. However, the long construction period, the high noise during operation, large dust and the fast deterioration rate of the skid resistance at the entrance and exit sections, which significantly influence the tunnel safety and environmental protection, have become a limitation for the further application of the cement concrete on the tunnel construction.

In recent years, with the increase of requirements for tunnel pavement safety, comfort and environmental friendliness, asphalt pavement has become more and more widely used in long tunnels, due to its low noise, low dust, easy maintenance and good comfort [1]. However, conventional tunnel asphalt pavements also cause significant safety and environmental concerns. At high temperatures and under the vertical and horizontal loads during the starting, accelerating, decelerating and braking, shear deformation distresses, such as rutting and upheaval, may occur on the asphalt pavements [2]. In the construction of tunnel asphalt pavement, a large amount of smoke, exhaust gas and dust will be generated. When a fire occurs in a tunnel, the combustion-supporting effect of the asphalt will increase the risk of tunnel fires, especially in long tunnels.

Recently, two main flame retardant methods haven been proposed. The first method is adding flame retardant, which mainly makes asphalt pavement achieve an ideal flame retardant effect. It mainly includes two forms: Directly adding flame retardant to asphalt or flame retardant mineral powder. The second method is gasoline escape, which makes use of the large void characteristics of the asphalt mixtures to make the gasoline escape quickly beyond the pavement, therefore, reduces the amount of gasoline involved in combustion, and achieves the purpose of flame retardant.

At present, most of the research on flame retardant asphalt pavement is to apply mature flame retardants in the field of flame retardant to asphalt. Usually, flame retardants are directly mixed with asphalt at about 160 °C to prepare flame retardant asphalt, and then mixed with coarse and fine aggregates, mineral powder and fibers to form asphalt pavement. Most of the research results of flame retardant asphalts are applied to asphalt felt and asphalt coating. Among them, Jolitz and Kirk [3] used organic bromide, potassium citrate, amine and other flame retardants; Walter [4] used borate flame retardant; Grube and Frankoski [5] used borate flame retardant; Brown et al. [6] used bauxite, brucite, etc.; Graham [7] used halogen flame retardant; Slusher et al. [8] used intumescent flame retardants. In order to determine the flame retardant and mechanism of asphalt synergistic flame retardant by decabromodiphenyl ethane (DBDPE) and Sb_2_O_3_, Zuo et al. [9] studied the effects of DBDPE and Sb_2_O_3_ on the flame retardant and thermal decomposition characteristics of SBS asphalt. The test used was based on the results of the limiting oxygen index test, the smoke density test, and the thermal gravimetric and differential thermal test. Cong et al. [10] explains the mechanism of asphalt burning and elucidates the different forms of flame retardants. Xu et al. [11] conducted horizontal burning, limiting oxygen index (LOI) and direct burning tests to evaluate the effects of magnesium hydroxide (MH) on flame retardancy for asphalt. Jia et al. [12] evaluated the effect of three different combinations of flame retardant additives on an asphalt binder flaming using the oxygen index test method. Zhao et al. [13] studied the flame flame-retardant asphalt for a tunnel containing various kinds of flame retardants, including decabromodiphenyl oxide (DBDPO), DBDPE, Sb_2_O_3_, ZnBO_3_, Mg(OH)_2_, and Al(OH)_3_ under different additive concentrations. Zhang et al. [14] conducted horizontal burning and limiting oxygen index (LOI) to evaluate the effects of mixed decabrombromodiphenyl ethane (DBDPE) and antimony trioxide (Sb_2_O_3_) on flame retardancy for epoxy asphalt binder. The influence of DBDPE/Sb_2_O_3_ on the rotational viscosity, the thermal and mechanical properties of epoxy asphalt binder was assessed by thermogravimetric (TG) analysis, differential scanning calorimetry (DSC), and a tensile test. Wu et al. [15] investigated the flame retardant mechanism of hydrated lime (HL) on asphalt mastics via a range of analytical techniques, including the cone calorimeter test, and thermogravimetry and differential scanning calorimetry analysis.

The structural performance of asphalt under heavy traffic loading is a vital aspect for pavement practitioners [16]. However, the effects of flame retardant have an unpropitious impact on the performance of the asphalt, and have not formed a special flame retardant system. There are still many problems, such as toxicity, smoke, and construction difficulties.

In recent years, the demand for functional pavements has kept increasing. New materials and technologies are being developed to improve the functionality of new roads. This innovative polyurethane thin overlay (PTO) was developed for the maintenance of existing roads and constructing new roads. The performance-related mechanical properties of the PTO specimens were evaluated with several preliminary methods in previous studies [17], and the basic strength of the specimen was tested through a uniaxial compression test. From this test, the PTO showed excellent mechanical behavior with roughly 6.6 GPa Youngs’ modulus. Furthermore, dynamic stability was evaluated with cyclic compression tests. It presented a superior performance in the long-term durability of the materiel, which was more than ten times higher than conventional porous asphalt. The fatigue resistance was assessed by the indirect tensile strength test. From which test, the strong inherency tensile strength was also examined. Porous PTO can also facilitate stormwater infiltration and increase the driving safety and maneuverability of automobiles [18,19,20,21]. Apart from mechanical properties, the hydraulic conductivity and the durability against clogging have also been verified much larger than conventional porous pavement materials, due to excellent void connectivity and pore structures [22].

Based on the previous study, the concept of PTO may be a feasible and effective way to enrich the innovative functions of tunnel pavement. In this paper, the research aims to evaluate the functional properties of PTO. Apart from conventional functionalities, e.g., skid resistance and drainage, some properties, such as noise reduction, solar reflection, and especially combustion properties, have aroused great interest in tunnels of metropolitan cities. A conventional asphalt (open graded friction course (OGFC) and stone mastic asphalt (SMA)) and concrete pavement materials were used as control materials. Compared with conventional tunnel pavement materials, significant improvements were observed in mechanical and functional properties, as well as environmental performance. Therefore, this wearing layer can potentially provide pavements with new eco-friendly functions. This study provides a comprehensive analysis of these environmentally-friendly materials, paving the way for a possible application in tunnels, as well as some other fields, such as in race tracks in stadiums.

## 2. Methods

### 2.1. Materials and Preparation of the PTO Specimens

To achieve a high permeability, a void-rich thin overlay, such as OGFC, is currently the most feasible and effective way [23,24]. Although potentially beneficial, permeable pavement materials face some challenges, due to the high-void content (less stone-to-stone contact regions) and the viscous nature of bitumen. In conclusion, the poor mechanical durability [25] and unfavorable clogging behavior [24,26] represent the main obstacle inhibiting a wide application of permeable pavements [27]. The latest research substituted conventional bitumen with bio-based polyurethane (PU) in order to create a sustainable permeable pavement. The bio-based polyurethane consists of various polymers that are synthesized by a poly-addition reaction of a di-isocyanate or a polymeric isocyanate with a polyol. Specifically, among the polyol components, traditional petroleum raw materials are replaced by organic oils. The synthesis is based on the connection of isocyanates and hydroxyl groups that lead to the formation of a urethane group [28]. Polyurethane elastomers consist of the polyol component and the isocyanate component. By modifying the components, a wide range of material properties, ranging from brittle to elastic, can be designed. Preliminary research conducted at RWTH Aachen University (Germany) suggests that PU-bound porous pavement structures exhibit high permeability, high strength, high resistance to permanent deformation and increased fatigue resistance [17,29].

The mechanical and morphological properties of aggregate are essential for the functional and mechanical properties of conventional OGFC [30,31,32]. Various investigations have focused on using natural or recycled aggregate in pavements [33].

**Figure 1:**
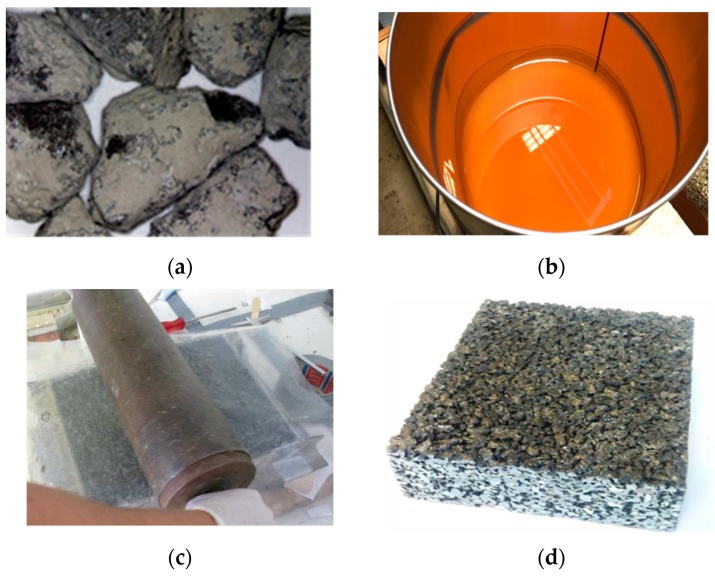
Raw materials for polyurethane (PU) specimens: (**a**) TiO_2_-coated aggregate; (**b**) Bio-based polyurethane binder; (**c**) Compaction process; (**d**) PU specimen.

In producing the PTO mixture, the conventional natural aggregate within the particle size range of 2.0–5.6 mm was replaced, and the remaining 30% was filled with natural sand (0–0.2 mm). A 2-component polyurethane product was selected as the binder for the PU specimens. The basic components of PU are shown in Figure 1.

**Figure 2:**
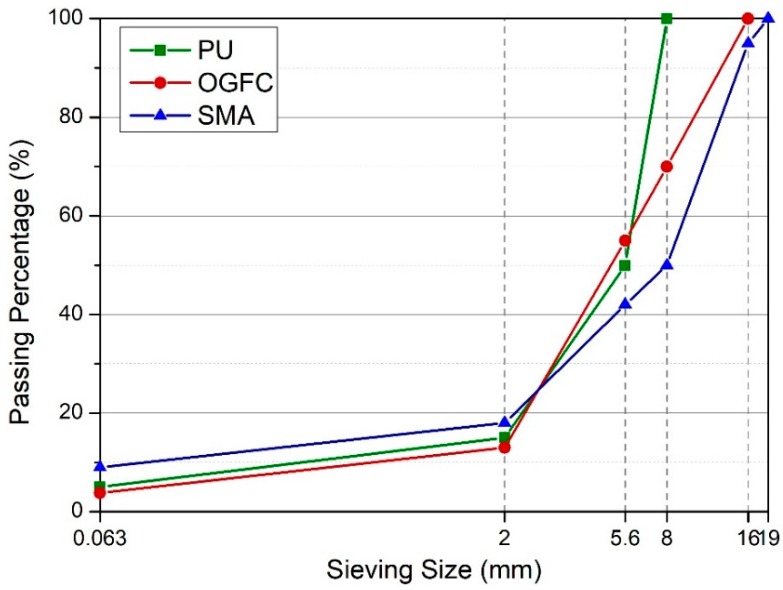
Grain size distribution of PU, open graded friction course (OGFC), and stone mastic asphalt (SMA).

Conventional OGFC, SMA and cement were selected as reference materials in this study. The conventional OGFC and SMA specimens were composed of crushed diabase aggregate, limestone powder, and a polymer modified bitumen binder. The mixtures were prepared by means of Marshall compaction (50 impacts per side). The grain size distribution and detailed mixture design of the PU, OGFC and SMA reference specimens were based on the porosity and the maximum density process, which are given in Figure 2 and Table 1, Table 2 and Table 3 respectively.

The preparation of different PU specimens followed a similar procedure to hot-mix asphalt (OGFC and SMA). However, mixing polyurethane can be conducted at room temperature, because the polymerization reaction and viscosity of polyurethane are not strongly affected by temperature. After the two components of polyurethane were thoroughly mixed, the binder is added to the aggregate. The components are mixed for a few minutes to obtain a homogenous mixture in which all surfaces of the aggregate are coated with binder. After mixing, a pre-determined amount of polyurethane-bound mixture is placed into a mold to obtain specimens of the desired bulk density. A heavy roller is used for compaction of the mixture. After approximately 24 h, the hardening process is completed, and the specimens can be removed from the mold.

Another reference specimen had a grade of C40/10 which is conventionally used in PCC pavements in Germany. Table 4 shows the batching proportions for C40/10 used in this study. The design slump values for PCC mixtures were 80.

### 2.2. Acoustic Performance

Existing studies have shown that tire-road noise mainly happens based on the inner resonance of the tire surface and pavement cavities (Pcavity), air flow around the vehicle body (Vehicle) and tire vibrations (Pvibration) [34]. Pcavity, which determines the noise absorption, is researched by a huge amount of research as the most important factor when evaluating the acoustic performance of the PU specimens [20,35].

**Figure 3:**
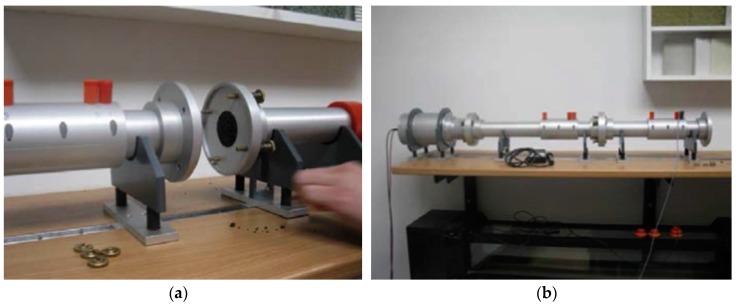
Installing the impedance tube for characterizing the noise absorption of PU samples: (**a**) Connecting the sample holders; (**b**) completely installed impedance tube device.

In order to examine the sound absorption capacity of pavement material, the impedance tube test according to DIN EN ISO 10534-2 was adopted, which has been widely applied for noise absorption evaluation in previous studies [36,37]. This test method uses an impedance tube, two microphones, an amplifier, and a recorder to evaluate the sound absorption coefficient (see Figure 3). Due to the shape of the tube, the sound waves propagate as flat waves inside the tube. A frequency sweep is generated and played back via the connected amplifier and loudspeakers. The generated sound frequencies are measured with the microphones installed at the tube. The acoustic transfer functions of the two microphone signals are used to calculate the reflection factor and the absorption factor at normal incidence and the impedance ratio of the test material according to DIN EN ISO 10534-2 (https://www.perinorm.com/document.aspx). All test samples, including PU, OGFC, SMA, and cement, were regulated in the same dimension of normal pavement samples with 100 mm diameter and 40 mm height, so that it corresponds to the practical layer thickness. There are three parallel test pieces of each variant; all specimens were measured three times each.

### 2.3. Reflection Test

The reflection rate is an indicator of how reflective a pavement is of solar radiant. Higher reflection rate means more radiant energy could be reflected back into the air, and pavement will absorb less energy. It has been reported that the reflection rate depends strongly on pavement surface color, service condition, and texture features. In general, the reflection rate of pavement falls in the range of 0.10–0.30 [33], with brighter pavements higher reflection rates. The reflection rate may be not a big problem under a tunnel section, but it is worthy of being investigated for the application of the innovative material in the entrance and exit sections of the tunnel, where is not sealed under the tunnel section. Furthermore, these results can offer comprehensive information and may benefit the readers who would like to apply the innovative material in other practical fields.

**Figure 4:**
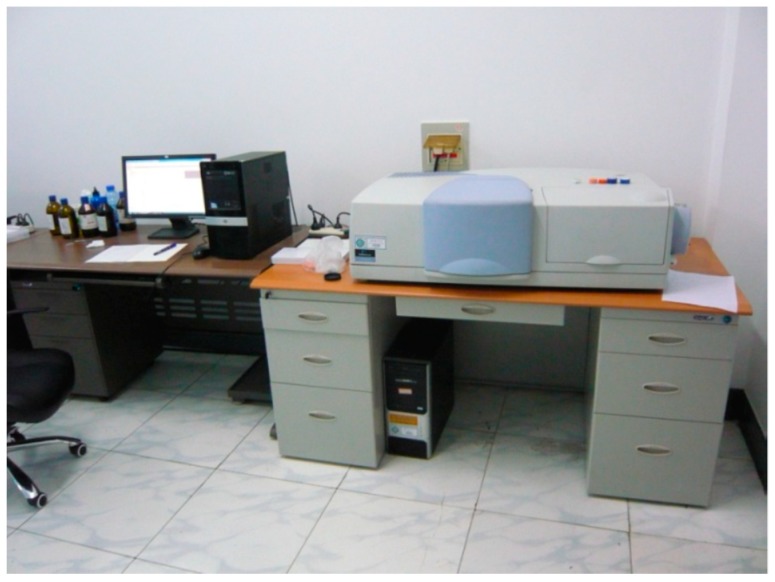
UV/VI/IR Spectrophotometer.

In this research, UV/VI/IR Spectrophotometer (see Figure 4), a device commonly applied to detect substance based on the absorption spectrum, was used to test the reflection rate of different samples. Samples were radiated by different lights within the wavelength range of 400–2000 nm with a step length of 5 nm, followed by detection of the reflected energy. Then, the reflection rate of different wavelength light was automatically calculated.

**Figure 5:**
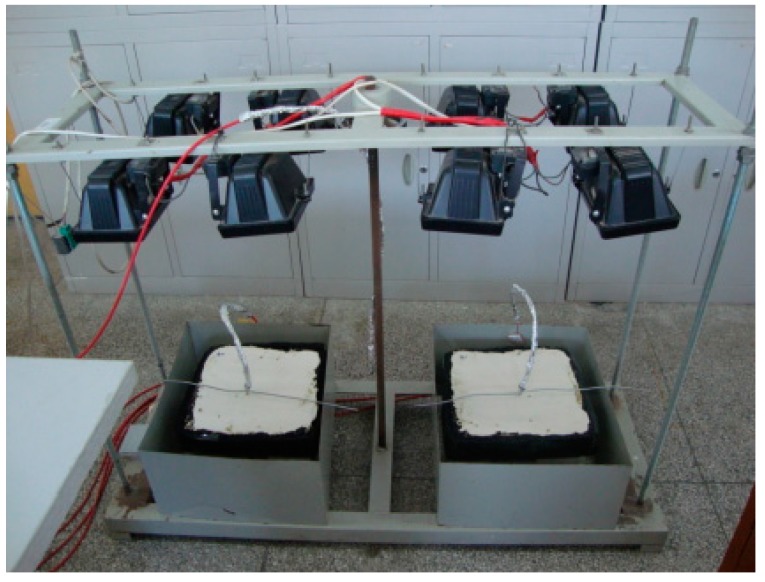
Simulation of solar radiation using an iodine tungsten lamp.

To further test the samples’ heat reflection, a specially-designed iodine tungsten lamp device (Figure 5), with a radiant energy of 820 W/m^2^, was used to simulate solar radiation. All samples were surrounded by thermal insulation cotton in the bottom and four sides. The temperatures were measured by a sensor placed 2.5 cm in the sample. The setup of this test was defined according to our previous research, which can efficiently test and evaluate the samples’ heat reflection in the laboratory. A calibration factor or shift factor which can convert the laboratory results to the field will be studied in the future.

### 2.4. Flammability Evaluation Method

**Figure 6:**
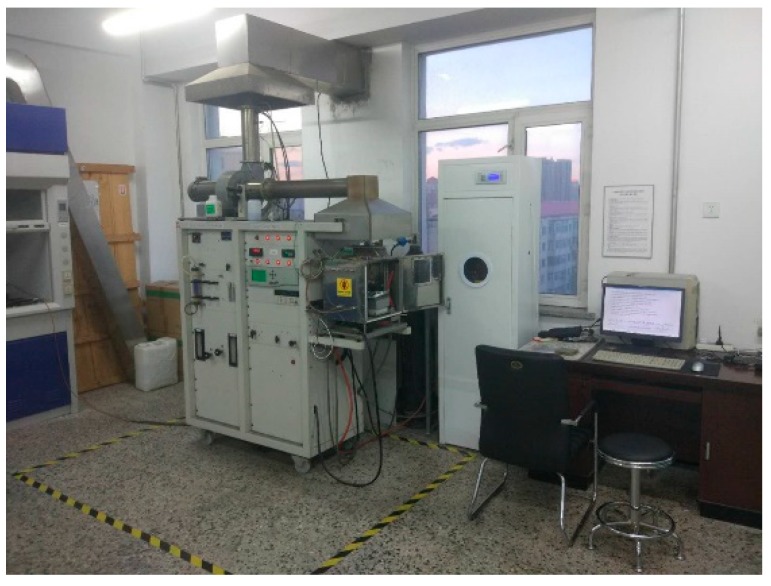
Appearance and components of the cone calorimeter.

The ignition of a vehicle is the main cause of the tunnel fire. However, for the pavement engineers, how to reduce the harm caused by the ignition of the pavement materials is the main concern. Especially, the smoke may be extremely harmful to the drivers trapped within the tunnel. Therefore, the combustion performance of the materials was evaluated in this study. The cone calorimeter was designed by Dr. Babrauskas of the National Institute of Standards and Technology (NIST) in 1982 based on the principle of oxygen consumption. It is a vital test instrument for evaluating materials’ combustion performance. As shown in Figure 6, the cone calorimeter is mainly composed of a carrier, a combustion chamber, a ventilation system, a flue gas measuring system and a gas analyzer.

The main test parameters it can include the heat release rate (HRR), total heat release (THR), effective combustion heat (EHC), ignition time (TTI), smoke and toxicity parameters, and mass change parameters (MLR). These parameters can be used to evaluate the combustion performance or flame retardancy of materials. Compared with the traditional test method, the cone calorimeter can get more data in one experiment. In addition, the combustion test environment of the cone calorimeter is similar to the real combustion environment, and the test results have a good correlation with the real conflagration, which has a good reference value for the evaluation of the combustion performance of materials.

In this test, it is required that the cross section of the tested specimen be 100 mm × 100 mm square, and the mass should not exceed 200 g. The experimental power of the cone calorimeter is 50 kW/m^2^, and the corresponding temperature is 780 °C. Before the test, the side and bottom of the sample were wrapped with aluminum foil, and then the measured sample will be forced to ignite under 50 kW/m^2^ thermal radiation intensity. The data obtained during combustion will be collected by a computer.

## 3. Results and Discussion

### 3.1. Results of the Acoustic Test

**Figure 7:**
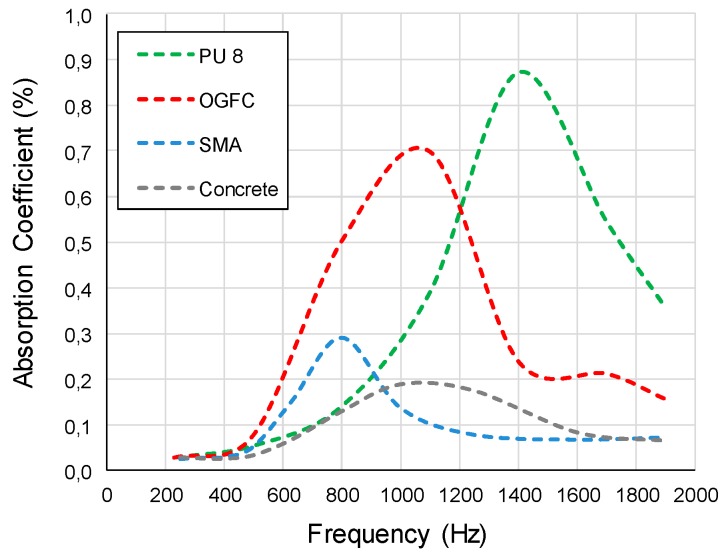
Acoustic absorption-coefficient curves.

The acoustic absorption coefficients of all specimens are shown in Figure 7. The figure indicates that the acoustic behavior of the four types of material is distinctively different. In general, the acoustic absorption properties of porous pavement material (both PU 8 and OGFC) are far higher than the conventional SMA and concrete specimens. In the relevant frequency range of 800–2500 Hz, PU specimens show a peak absorption coefficient of about 84% at 1400 Hz, whereas the OGFC specimens show a peak absorption coefficient of about 70% at 1000 Hz. The SMA and concrete only show a relatively lower peak noise absorption about 30% and 20% at 800 and 1080 Hz respectively. However, the noise frequency near pavement is usually in the range from 1200 Hz to 1600 Hz if the travel speed around 70 [34,36]. In which case, the PU material can have the highest noise absorption property among all materials. The peak noise absorption frequency is a function of the sample height and the pore structures. During the test, the specimens of PU, OGFC, SMA and concrete were kept in the same height of 4 mm. In this case, the difference can be mostly attributed by the porosity and pore structures. The absorption coefficients of PU samples surpass that of the other samples throughout the entire frequency domain. In comparison with the conventional OGFC, the PU exhibits higher coefficients of absorption across a wider range of frequencies, rendering it a material with far superior acoustic properties. The excellent noise reduction ability is mainly due to the large connective void content within the PU, which can expand the frequency range of absorption. Therefore, the PU material is the most suitable for the application in tunnel pavement, due to the relatively high noise frequency level.

### 3.2. Results of Heat Reflection

**Figure 8:**
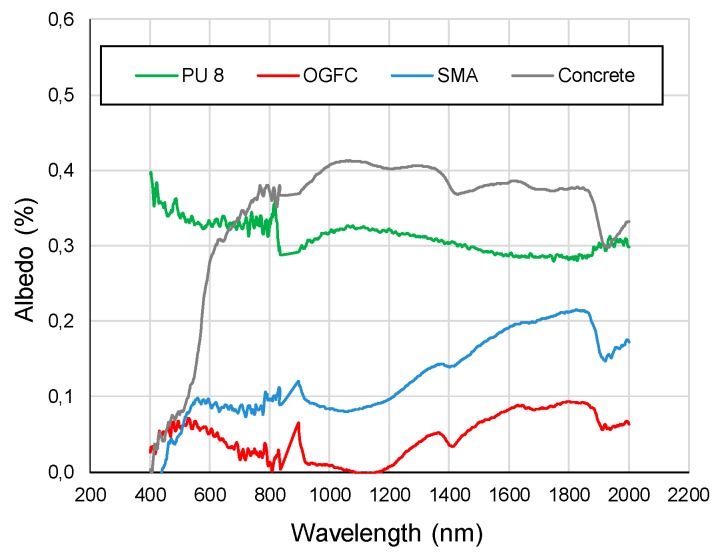
Simulation of solar radiation using iodine tungsten lamp.

Figure 8 presents the results of the heat reflection tests subjected to light in the wavelength of 400 to 2000 nm. It can be observed that the light reflection rate varied significantly among different surfaces. Freshly manufactured asphalt, which is black, has the lowest light reflection in the whole range of wavelength. In other words, the majority of solar radiation will be absorbed by asphalt, thus increasing the road surface temperature. In which case, the OGFC and SMA, that use bitumen as a binder material, exhibit lower heat reflectance values, which in most cases is less than 0.1%. The surface temperature of OGFC and SMA is also relatively high, reaching 74 degrees after 6h solar radiation.

In contrast, PU and granite have the highest light reflection rates in wavelength rage of 800 to 2000 nm. PU experiences a continuously upward trend in terms of heat reflection and eventually reaches a peak at 2000 nm, with a reflection rate of around 0.5. Infrared mainly contributes to the most thermal effect of light, especially in practice. The infrared wavelength is in the range of 760–2500 nm, which falls into the highest reflection wavelength range of PU and granite. In this case, PU and granite in the actual application of heating rate are lower.

**Figure 9:**
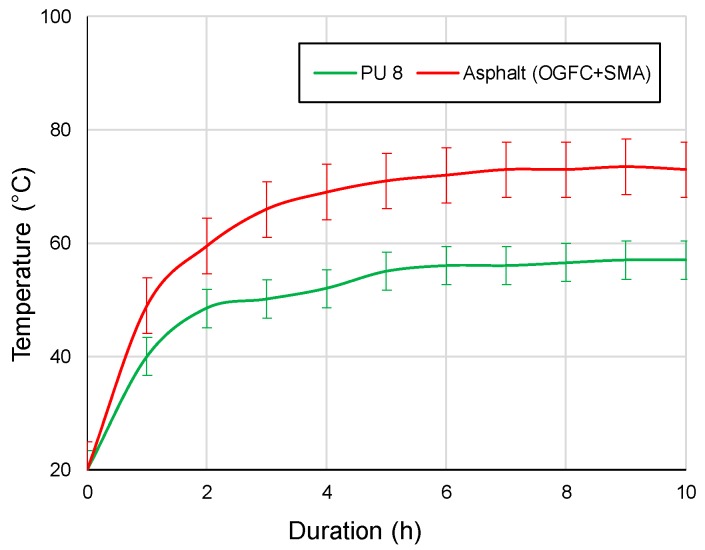
The different reflection results of a heat reflection surface.

When exposed to the same simulated solar radiant energy of 820 W/m^2^, the surface temperature of PU is lower than that of normal asphalt surface during the testing period (see Figure 9). After about five hours of heating, the temperature of both samples becomes steady. The eventual temperature of PU was 20 °C lower than that of the normal asphalt surface after ten hours of heating. The result indicates that PU, which is brighter, provides higher heat reflection rate in comparison with the black normal asphalt surface, which significantly reduces the pavement surface temperature. Therefore, this innovative pavement surface treatment method can potentially lessen the urban heat island effect.

### 3.3. Results of Combustion Tests

#### 3.3.1. Ignition Time (TTI)

TTI is an important parameter for evaluating the combustion properties of materials. It refers to the time spent from heating the surface of materials to continuous combustion at a preset incident heat flux, and its unit is second. It can be used to evaluate and compare the refractory properties of materials. The longer the TTI is, the harder the material is to ignite under the specified experimental conditions.

**Figure 10:**
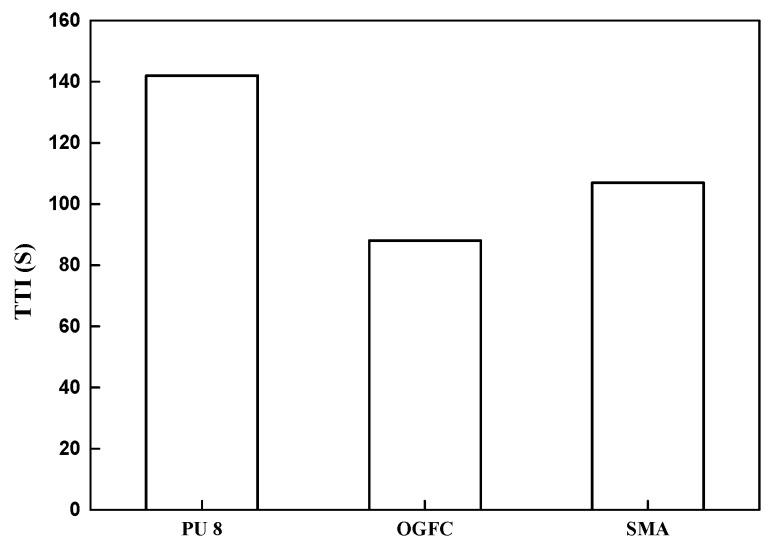
Comparison of ignition time (TTI) among PU, OGFC and SMA.

As shown in Figure 10, the TTI of OGFC is smaller than that of SMA. This is because the air void of OGFC is larger than that of SMA, resulting in the larger exposed asphalt area and air contact area, thus it is easier to ignite. The TTI of porous polyurethane mixture is larger than that of OGFC and SMA, indicating that PU is more difficult to ignite than asphalt. Because there is no obvious ignition phenomenon of cement, it is regarded as incapable of ignition, and its TTI is not shown in the figure.

#### 3.3.2. Heat Release Rate (HRR)

HRR refers to the heat release rate per unit area after the material is ignited under the preset radiation intensity, and its unit is kW/m^2^. The maximum value of HRR is the peak value of the heat release rate (pkHRR). The peak value indicates the maximum degree of heat release during combustion. The greater the HRR and pkHHR are, the greater the heat released from the burning of the material, and the greater the fire hazard.

**Figure 11:**
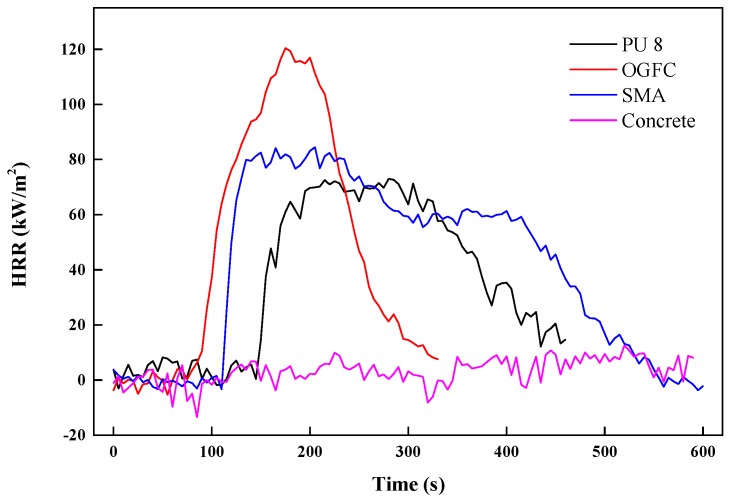
Comparison of heat release rate (HRR) among PU, OGFC and SMA.

Figure 11 shows that the heat release rate curve of OGFC, with the highest pkHRR of 120.38 kW/m^2^, is relatively left-centralized, meaning the quickest complete combustion and the greatest risk of fire. The HRR curve of SMA, with the second largest pkHRR, basically encloses that of PU, which indicates that SMA emits more heat per unit time and the heat release of it lasts for a longer time than that of PU. By comparing the curves of PU, SMA and OGFC, it can be seen that PU has better flame retardancy than asphalt. Finally, the HRR curve of cement fluctuates at 0, indicating that the cement will not be ignited.

#### 3.3.3. Total Heat Release (THR)

THR refers to the sum of heat released by materials from ignition to flame extinction at a preset incident heat flux in the unit of MJ/m^2^. Combining HRR with THR can better evaluate the combustibility and flame retardancy of materials, which has a more objective and comprehensive guiding role for fire research.

**Figure 12:**
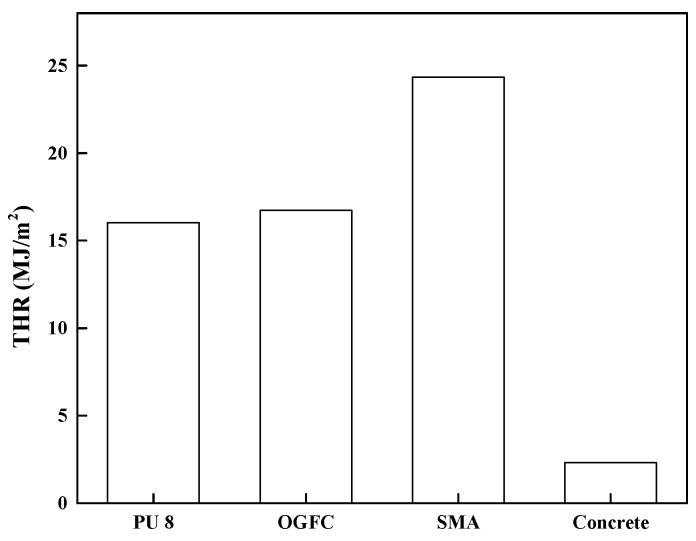
Comparison of total heat release (THR) among PU, OGFC and SMA.

Figure 12 shows that SMA has the largest THR of 24.34 MJ/m^2^, because it has the largest asphalt content. Besides, the THR of OGFC is 16.73 MJ/m^2^, close to that of PU of 16.04 MJ/m^2^, but the ignition time of OGFC is small, and the heat release of it is concentrated and intense. Therefore, asphalt pavement is more dangerous than polyurethane pavement when they are on fire. Finally, the total heat released of cement is very small but still exists, because the cement has only seven days age and has not been fully hydrated.

#### 3.3.4. Specific Extinction Area (SEA) and Total Smoke Release (TSR)

SEA is a dynamic parameter to characterize the amount of smoke emitted at every moment in the combustion process, which can reflect the ratio of volatile matter per unit mass to smoke (unit: m^3^/kg), while the TSR can reflect the total amount of smoke generation and release per unit area in the fire field (unit: m^3^/m^2^). These data have a good correlation with the smoke parameters of large-scale experiments.

**Figure 13:**
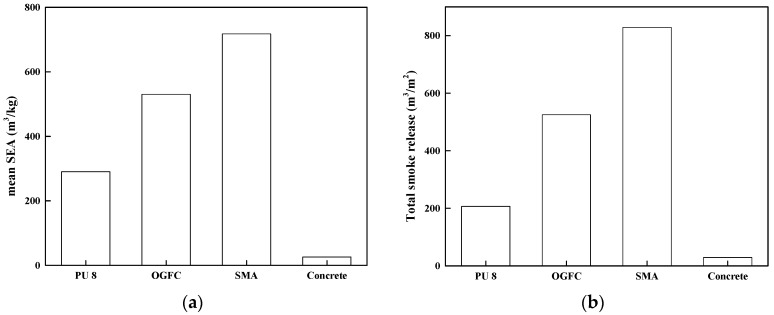
Comparison of the results among PU, OGFC and SMA. (**a**) mean for the specific extinction area (SEA); (**b**) total smoke release (TSR).

Figure 13 shows that SMA has the largest average smoke emission and total smoke emission, followed by OGFC, PU and cement. In fires, excessive smoke may lead to asphyxiation, hypoxia and death. Therefore, asphalt pavement is more harmful to the environment and the human body than polyurethane pavement when burning.

#### 3.3.5. Fire Proceeding Index (FPI)

FPI combines ignition time and peak heat release rate, and is the ratio of ignition time to peak heat release rate (unit: s*m^2^/kW). The larger the FPI value, the stronger the fire resistance.

**Figure 14:**
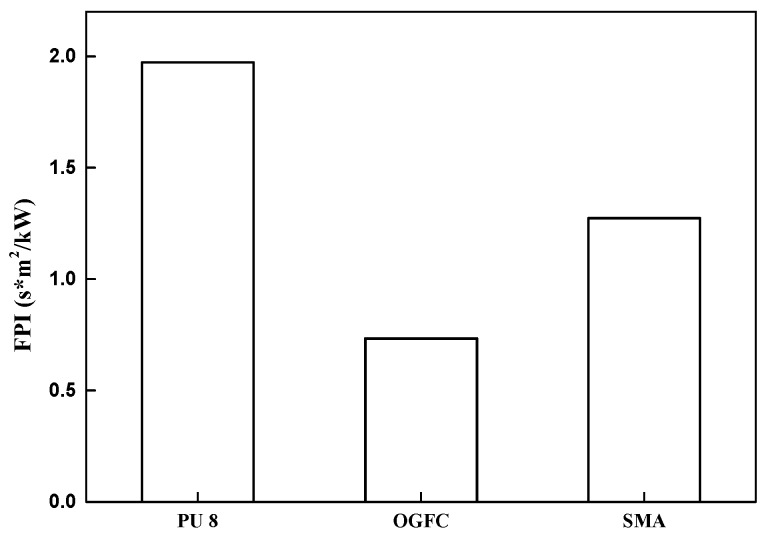
Comparison of fire proceeding index (FPI) among PU, OGFC, and SMA.

Figure 14 shows that the FPI of OGFC is smaller than that of SMA, and the FPI of asphalt mixture is smaller than that of polyurethane mixture, which indicates, as previously analyzed, that polyurethane pavement has better flame retardancy than asphalt pavement. Besides, the pkHRR of cement is almost zero, so its FPI value is extraordinary and not shown in the figure.

## 4. Summary and Conclusions

In this paper, the functional properties of PTO were evaluated, and compared, with the conventional asphalt (OGFC and SMA) and concrete pavement materials regarding the noise reduction, solar reflection and combustion properties. Significant improvements were observed in functional properties, as well as environmental performance. The following summarizes the main findings of this study:

- Based on the evaluation by the acoustic tube test, both PU and OGFC show superior acoustic properties in comparison with conventional SMA and concrete pavement materials. However, comparing to OGFC, PU has a wider range of noise absorption. Especially for high frequency noise, which more likely exists in the tunnel, PU exhibits a maximum noise absorption coefficient, while other materials have almost no noise absorption within this range. Concluding, PU is more efficient in noise reduction of tunnel pavement.
- The light reflection rate varied significantly among different pavement surfaces. Based on the heat reflection tests, the OGFC and SMA which use bitumen as a binder material, exhibit the lowest heat reflectance values. The concrete presented the highest reflection rate and followed by PU in wavelength it ranged from 800 to 2000 nm. On the other hand, an increase of the radiation time results in a significant increase in the surface temperature of the PU and asphalt material. However, compared to the asphalt material, the increase of temperature on the PU surface is almost 30% less. Therefore, this PU pavement surface can potentially lessen the urban heat island effect. It is meaningful when the PU is applied in the entrance and exit sections of the tunnel, where it is not completely sealed under the tunnel section.
- By comparing the results of the combustion tests, it can be seen that PU has a better flame retardancy than asphalt (OGFC and SMA). Particularly, the TTI of PU is larger than that of OGFC and SMA, indicating that PU is more difficult to ignite than asphalt. Asphalt (OGFC and SMA), with the higher pkHRR than PU, can result in faster combustion and a greater risk of fire. SMA has the largest THR, indicating that the largest amount of heat is released. The THR of OGFC is close to that of PU, but the ignition time of OGFC is small, and thus the heat release is more concentrated and intense. SMA has the largest average smoke emission and total smoke emission, followed by OGFC, PU and cement. FPI of asphalt is smaller than that of PU.

Overall, this study has demonstrated that this innovative wearing layer can potentially provide tunnel pavements with new eco-friendly functions compared with the conventional asphalt materials. In further research, the PTO will be applied and analyzed in the construction of model tunnels. The relative production and laydown direct cost relation for all the mixtures will be investigated in the next step. More comprehensive tests will be carried out to investigate the mechanical and functional properties of the PTO materials, i.e., long-term performance of PTO under repeated dynamic loading (traffic loading in the field) needs to be investigated carefully through dynamic fatigue and fracture tests [38]. The possibility of the PTO applied in race tracks in the stadiums will also be investigated.

## Figures and Tables

**Table 1:** Mix design of PU mixture.

PU Materials	Grain Size (mm)	Mass Percentage (%)	Apparent Density (g/cm^3^)
Limestone	0−0.063	5.0	2.820
Diabase	0.063−2	15.0	2.850
	2−5.6	52.0	2.850
	5.6−8	100.0	2.850
Polyurethane	2-component polyurethane, 6.5 M.−%		1.09
PU Mixtures	Air void content 28.9 Vol.−%		1.93

**Table 2:** Mix design of OGFC mixture.

OGFC Materials	Grain Size (mm)	Mass Percentage (%)	Apparent Density (g/cm^3^)
Limestone	0−0.075	4.0	2.820
Basalt	0.075−2.36	10.2	2.820
	2.36−9.5	62.3	2.820
	9.5−16	23.5	2.820
Bitumen	Polymer modified bitumen 40/100–65 A, 4.5 M.−%		1.472
OGFC Mixture	Air void content 21.2 Vol.−%		2.090

**Table 3:** Mix design of SMA mixture.

SMA Materials	Grain Size (mm)	Mass Percentage (%)	Apparent Density (g/cm^3^)
Limestone	0−0.075	10.0	2.820
Basalt	0.075−2.36	9.5	2.820
	2.36−9.5	35.5	2.820
	9.5−16	40.0	2.820
	16−19	5.0	
Bitumen	Polymer modified bitumen 40/100–65 A, 5.4 M.−%		1.472
SMA Mixture	Air void content 3.1 Vol.−%		2.471

**Table 4:** Mix design of the cement (kg/m^3^).

Cement	Pulverized Fuel Ash	20 mm	10 mm	Fines	Water	Aggregate Cement Ratio	Water Cement Ratio
330	110	725	345	620	185	3.84	0.42
